# The Outlook of Medicine and Surgery

**Published:** 1883-02

**Authors:** J. McF. Gaston

**Affiliations:** Campinas, S. Paulo, Brazil


### ATLANTA MEDICAL REGISTER.

VOL. II.] FEBRUARY, 1883. [No. 5

### Original.

### THE OUTLOOK OF MEDICINE AND SURGERY.

BY J. McF. GASTON, M.D., CAMPINAS, S. PAULO, BRAZIL.

The present status and future prospects of the medical profession should interest all of its members.

Among the necessary evils of human society, there appeared in the early periods of civilization certain persons who were recognized by various names with the prerogative of curing diseases; and in these latter days, this class has multiplied so as to occupy a prominent place among the movements of the world. With the refinements of progress, mankind have become subject to more frequent and more serious disorders of the physical organization than were manifested under a ruder mode of living, and the demand has been correspondingly increased for medical men. The problematical question as to the sum total of good or evil resulting from the application of remedies for the cure of diseases has been resolved by the almost universal voice of the human race in favor of medication in cases of sickness, and hence the humane and philanthropic determination of a considerable portion of the human family to become acquainted with the most effectual mode of using medicine under the varying types of departure from the standard of health. That all do not seek the medical profession from sympathy for the sufferings of their fellow-beings, and that self-interest should be the moving principle in selecting this field of labor on the part of a large number, may be taken as exceptions to the general rule that those who look forward to the practice of medicine are actuated by a laudable desire to be serviceable to mankind.

The interchange of ideas among medical men and the accumulation of experience, with the organization of schools and other associations, have combined to elevate medicine. The progressive spirit which has induced the medical profession to abandon those modes of treatment for diseases which were found insufficient, and to adopt those which have secured satisfactory results, indicates more independence of thought among practitioners at present than at any former period of the history of the healing art within our knowledge.

In view of this substitution of rational experiment for routine, it is found that investigation with observation of facts, as to the cause, nature and consequences of disease, in connection with the means of relief, tends to the correction of false theories and blind impiricism in practice.

The advancement of knowledge by statistics in all the clinical departments and the accurate results obtained by the skillful use of the microscope serve to redeem the profession from the domination of dogmas and elevate it to a recognition of principles, so that medicine can no longer be classed as an art only, but is entitled to be considered a science. In this aspect of the aim and reach of the practice of medicine and surgery in all their extensive ramifications, it behooves us to appreciate correctly all the elements which go to make up the requirements for the proper discharge of these responsible duties, and to estimate critically the qualifications of the practitioner in each of these branches of the profession. It may not prove unprofitable, therefore, to direct the minds of those who are pressing forward in the ranks, to the conditions essential to success in their various fields of service. The shortcomings of considerable observation in a wide field of experience, during an active life, devoted to a general practice in medicine, surgery and obstetrics, will be excused by my colleagues, in attempting to point out some phases of the qualifications requisite for the true disciples of Æsculapius at the present day.

The prime consideration for all who enter the profession is a hearty consecration to its duties, and a profound conviction of fitness to undergo the trials incident to the faithful discharge of its obligations. He who would undertake its responsibilities without having his heart in the work, and simply as a means of gaining a livelihood, is destined to regard practice as a drudgery, and, instead of undergoing its privations and sacrifices in the spirit of a devotee, he must become a martyr to his inconsiderate zeal. When the office is degraded to that of a trade, in which the incumbent proceeds upon the principle that the laborer gains his pay by the sweat of his brow, the practitioner becomes merely a hewer of wood and a drawer of water to others, and soon regards himself as the servant of most exacting masters. The unreasonable demands which the sick and those around them make upon the medical attendant require a patient resignation, which perhaps is not equaled in the self-sacrifice of any other avocation in life, and he must bear and forbear in all things. The tacit compact between the physician and his patients, that he is held subject to their calls upon him, at all times and under all circumstances, must be fulfilled, let the sacrifice of ease be what it may to him. He must rise from his bed by night to make a long journey over bad roads if some one is unable to sleep and gets nervous from dragging through the cheerless and lonesome watches of restlessness. He must sally forth in the pelting storm when an anxious sufferer considers present comfort as of more importance than a regard for his health and well-being. He must cut loose from his family and friends when recruited for social enjoyment. In a word, he must forego all his personal preferences to satisfy the demands of the sick, let them come as they may to interfere with his plans. Any temporary delay, from indisposition of himself or family, is never accepted as a valid excuse, and it may be quietly hinted to him that another can be found if it does not suit him to respond promptly to all calls, in season and out of season. He is expected to watch at the bedside of a delirious patient when those who should take charge are sleeping soundly in an adjoining room, and to administer remedies by his own hand when it could be as well given by another. His time and energies are taxed to the uttermost, and yet he is expected to inform himself of all the most recent advances in his profession, and to be supplied with the latest improvements in instruments and in surgical appliances of all kinds, so as to be ready to meet every emergency which may come up. Not only must he make sacrifices in his compliance with the wishes of everybody by his active service, but he must passively submit to the narratives of all their ills, imaginary or real, with which they have been afflicted from their youth up, though it may have no connection with the desire to be treated. He must solve their doubts as to the propriety of this or that mode of living, to find at last that they take their own course as to their habits and customs. He must make the most of the good advice of his most devoted friends as to the management of his own domestic affairs, to realize that in the end they proceed upon the principle that every one takes care of himself in this world. It is especially incumbent upon him not to require payment of his accounts before it is convenient for his patrons to have money in hand over and above all other demands upon them ; and then, without any reference to the relative amount of service rendered, his bill must not exceed that of some other doctor that had practiced in the family during former years with great satisfaction to all concerned.

All in all, the medical man becomes the servant of servants, and has no independence of action in any matter that may interest his peace and quiet. He is the slave of the whims of unthinking men, women, and children ; and must go at the beck and call of all who imagine that they have a claim upon his very special regard. His face must be lit up with a smile of joy at meeting the gay, and his eyes must be filled with a tear for the distressed and yet abide his lot.

Not that we are called upon to submit to any kind of indignity, for this should be promptly resented by every member of the profession, coming from whatever source, high or low, rich or poor, white or black, strong or weak, as may be the individual who is guilty of such outrages. But it falls to the lot of the medical man to encounter annoyances from the weaknesses of persons, with no malice or ill will in their bearing, to whom the greatest indulgence is requisite, and this is not always limited to the sick, but is found on the part of those bound to the sufferer by the tenderest ties, who sometimes allow their affectionate interest to get the better of their prudence so as to do and say things which require our most charitable construction. One who cannot place himself in sympathy with such demonstrations should not engage in treating the sick. Though he may discriminate well in diagnosis, and prescribe judiciously for the disease, there is still much to be effected in becoming all things, to all men, and especially to all women, who are under his professional care. It is frequently our duty to overcome the unfounded prejudice of a patient or some friend of the same, in regard to a mode of treatment that is decidedly indicated in the given case, or to induce acquiescence in the performance of a surgical operation to which there is a natural repugnance, and much tact is sometimes requisite to attain the result which is essential to the safety of the patient or the reputation of the attendant. The sense of responsibility for the welfare of those entrusted to our care must lead to the use of all available means for correcting a disordered organization and we cannot altogether ignore the whims of people in dealing with their varying mental states under the influence of disease.

There are two extremes in treatment, which may unfit an individual for a faithful performance of his obligations to his patients—being on the one side callous to all suffering and on the other oversensitive to the pain and distress which seeks relief at his hands.

Bishop Butler, in his analogy, has placed man's emotional development on the true practical basis in declaring that as passive impressions lessen, active propensitives strengthen, and thus it turns out with the well regulated mental organization on the part of the medical attendant, as he comes to feel less for the sufferer he becomes more efficient in conferring benefits by his treatment. This is the law of habit and is very important in its practical working.

If it were otherwise and we became more impressionable as we come more frequently in contact with the manifestations of pain, the emotional excitement would unfit us for extending assistance to the sufferer, but owing to this fact that familiarity with disease increases the capacity for relieving the ills to which flesh is heir, the medical man should with less feeling render more efficient service to the sick.

It is not simply that his larger experience in his work fits him better for the discharge of his duties, but there is a principle underlying the intellectual constitution by which his natural powers become strengthened for the prompt execution of measures of relief. The nervous system is however in some persons so exciteable as to incapacitate them for the calm and deliberate exercise of their faculties under emergences, and such an individual can never become accustomed to scenes of blood or circumstances of great distress; being thus entirely disqualified to assume the responsibilities of a general practitioner. It might be that such a one could succeed as a specialist in some branch which would not tax his keen susceptibilities; and the fields of duty are so separated at the present day, that it is not a difficult matter to make a selection which may be congenial to the taste and temperament of any one who desires to devote his energies to some department of practice, to the exclusion of others. In the event that difficulties should present themselves in the experiences to be undergone in all other professional service, the delicate and exacting operations of the oculist offers an ample and lucrative field of labor for a specialist, and does not excite that repugnance which a sensitive constitution encounters in almost all other branches of practice. The combination of the diseases of the ear, nose and throat constitutes another interesting specialty for those who are by nature unfitted for the grave and more serious undertaking of a surgical character. The multiplicity of disorders to which these parts are subject and the facility with which the sufferers may be brought together from remote parts, enables one who limits his attention to this sphere of duty to be fully occupied and to reap satisfactory results in all respects. It is held by some, and with some show of reason, that there is a tendency to dwarf the mental powers in being thus devoted to one single department, yet the perfection attained in the details of a specialty compensates for any supposed influence of this kind, and moreover the scope of investigation extends itself into such minutiæ, when one's attention is fixed upon a special topic, that thought is varied greatly.

If we consider the extensive range of observation that must be made to comprehend all that pertains legitimately to the sphere of the oculist it must be seen that there is no pent up utica that contracts his powers, but he has the whole vast field of light and the laws which control its manifestations to occupy his mind and afford ample opportunities for exercising all his faculties.

Those who have concentrated all their energies upon the treatment of some particular disorder of the human organization, still find in its correlations with other parts, sufficient to gain various activity to the intellect, and even the cancer doctor who thoroughly investigates every thing associated with this depressed condition has a wide range for exploration.

It is an indubitable fact that advancement in the sum total of medical knowledge has been materially promoted by the division of labor which has ensued from the special consideration bestowed upon distinct branches; and it would not have been possible for any general practitioner to accomplish what has been attained by specialists in the various departments of investigation. Indeed the growing tendency of the last decade has been to have the labors of scientific experimentalists independent altogether of the practical duties of the profession, and it is thus that the vast progress of the age has come to be recognized throughout the world.

Yet there must continue to be many who will undertake the responsibilities of family practice with all its worrying cares and vexations.

Self possession must be maintained amidst the uproar of an anxious household, who are full of grief and alarm over the danger that seems to threaten one of the family. For instance a father comes running to your office with disheveled hair and glowing eyes, ejaculating in broken accents between his hurried inpsirations that his child has a fit and that he is afraid it will die before you can reach it. He urges you most pathetically to hurry up, and should it occur to you to look up a vial of hartshorn or ether to take along, he, insists that his darling babe requires your immediate presence. As you start out of the door he runs ahead calling in piteous tones upon you to come on with all possible haste, and having reached his residence in advance he rushes forward to find the little sufferer still writhing in convulsions, and returns to the door wringing his hands and uttering wild exclamations of grief while he summons you anxiously to proceed forthwith to the sick room. There all is found in confusion, the mother is crying over the child; a sister has collected all her smelling bottles and perfumery, a brother is running to and fro wishing to do something and, not knowing what to take hold of first, puts all around the room topsy turvy; a servant comes blowing with a large basin of water hot enough to scald the hair off a pig; the younger children stand in mute astonishment circling the sick child and can scarcely be made to budge at your approach. And now the joint cry of the parents comes, "for mercy's sake do something quickly." If you are sufficiently calm to inquire into the previous history of the case, what has been the state of the alimentry canal, what the child has eaten etc., no satisfactory answers are given, but on the contrary perhaps an old nurse interposes and commences a long harrangue upon the gradual development of some supposed disease from the falling of the navel or the appearance of the first tooth, or recounting its cries after making water, or attacks of a colicky pain from something that the mother ate three months ago, with many other problematical suggestions which have no bearing upon the present condition of things as regards treatment.

Should the patience of the medical man, ushered perhaps for the first time into this house under the emergency, not be exhausted by the wild frenzy of the family, he may have it further taxed by an old woman or two, who have come in to lend a helping hand and to volunteer their counsel to all concerned, who interpose a cautious demurrer to adopting what is recommended by the new comer and make sundry propositions to do this, that, or something else, which another doctor had resorted to, in such and such a case, within their knowledge or hearsay.

Do not suppose that all the trouble ends with this, as during the hubbub, to save time, the father has been dispatched with a verbal message to the nearest druggist to bring some ipecac and mustard, in place of which he asks for jalap and flax-seed, or some other articles quite as foreign to the order made.

Thus without personal composure, and control over those around you, under such circumstances, all are involved in confusion; and the medical attendant failing to make himself master of the situation fails to accomplish anything satisfactory to himself or the family.

A physician should possess that quality which in common parlance is defined as level-headed, and must keep his wits about him under every sort of disturbance; and he should seek to adapt himself to all classes and conditions of people and act as if to the manor born.

The incidents of medical experience are more strikingly varied than the observations of others engaged in the active pursuits of life, from the necessity of associating with all classes of human beings, in the discharges of professional services at all hours and under the peculiar circumstances which surround families in their privacy. The physician sees life as it is, and not as people would have others to believe it is, and mankind are presented to him in their every-day clothes instead of being arrayed in holiday garments. The diseases of his patients are not the only weakness which becomes patent to the medical attendant of a family, and it is incumbent upon him to use great circumspection in his relations to the members of the household so as to avoid any exposure of facts which should not be known outside of their immediate circle. There may be scenes of domestic trouble and suffering exhibited to him in his confidential visits, that no other human being would ever be allowed to witness; and without any injunction of secrecy on their part they must be guarded sacredly from the knowledge of those people with whom he comes in contact, so that not even the partner of his bosom may ever know what should not be known to others.

A physician may be ushered into the privacy of a family in great confusion, when a member is taken suddenly ill. When people make the most noise, there is not always the most danger, and it is a duty to them as well as to the high calling of the profession to maintain our self-respect by keeping cool even when every one around is excited. It sometimes occurs that embarrassments of a very different nature are encountered especially by a young unmarried practitioner, who is called to give advice in regard to the special derangement of the monthly discharge in the case of a sensitive daughter of a mother who has not learned that true modesty consists in calling things by their right names when facts must be known. While all appearance of rudeness must be certainly avoided in our approaches to the female sex under any circumstances, yet plain, direct interrogations are at times essential to a proper understanding of what is to be corrected, and we should try to impress upon our female patients the importance of giving distinct and unequivocal answers to our inquiries. To beat around the bush, and neither be understood, is the greatest prudishness, and the medical man who intends to do his duty to those who seek his advice should cultivate the habit of mutual frankness between himself and his female patients. It will often occur that in those suffering from uterine disease an ocular or digital examination is requisite for proceeding satisfactorily with the treatment, and under such circumstances the necessity of this step should be stated, and a compliance urged with due consideration and yet with firmness, so as to overcome the natural repugnance to such explorations.

Another scene involving the practitioner in still more serious embarrassment occurs in the chamber of the parturient female when the complications demand operative interference either with the hand or by the aid of instruments. It is generally found that the midwife has delayed to call a medical man and sometimes when called he relies too long upon the powers of nature from want of practical experience in the management of cases of difficult labors, so that in the end an accoucheur comes with instruments to discover that his colleague in attendance has got flurried, and upset the patient as well as her friends, with the urgent demand for immediate relief. A short time since I was summoned to apply forceps in an important case having two colleagues in attendance, one of whom met me at the door and after a hurried narrative of the condition of things, ushered me into the room of the parturient, when the husband, who was a man of education and more than ordinary intelligence, insisted that there was no time to be lost, as his wife was in a critical state. Having passed through a chilling atmosphere outside, warm water was asked for to restore the natural temperature to my hand, before making an examination, which impressed those around as an undue arrest of the precipitancy of action which was anticipated. Being satisfied of the nature of the position, the region of the bladder was deliberately examined, to determine as to the indication for using the catheter, previous to applying the forceps. The patient was then ordered to be placed in a transverse position upon the bed, with the breach near the margin, and assistants securing each foot. The blades of the instrument having been placed in a basin of warm water previously were now well oiled as well as the hand which was to guide the respective blades in their introduction. The great anxiety of the patient to terminate her suffering was manifested at every stage of the operation, and when traction was commenced she insisted that extraction should be effected forthwith, which of course could not be done, and she was enjoined to have patience. With her alternate rebuke to the attendant who preceded me and criticism upon my slow proceeding, I went on the even tenor of my way until all was concluded satisfactorily in the birth of the child. It being asphyxiated, as is usual in such cases, I did not accede to the suggestion of the midwife, backed up by the voice of her medical attendants, to divide the cord in which pulsation was distinct, but proceeded with artificial respiration until the lungs began to act, and after the child had cried lustily it was separated. After finding that the uterus contracted regularly, with some slight assistance the placenta was dislodged, and a bandage being placed around the abdomen the patient was made comfortable in bed; while I had the comfortable reflection that the surrounding commotion had not in the least modified the programme on my part. This being a family of wealth and of high social position, there was greater liability to be influenced by the exactions imprudently made for precipitation; and the medical man has to resist this tendency to yield to the demands of those of importance in society, when perhaps he would not experience like embarrassment in opposing the wishes of the humble. There are no considerations connected with the rank in social positions of the sick which should influence the medical attendant to depart from the standard recognized in the ordinary management of those under his charge. He shonld forget for the time all distinctions and proceed with his treatment in case of the most important personage in the land, just as would be proper for the humblest member of society. We read of a king having called a medical man, who was reported to have been successful in his general practice among his subjects, and he enjoined upon him at the outset that his case should be divested of all royal associations, and treated as if he were one of the common people of his dominion; thus recognizing the very erroneous proclivity of the profession to extend a different consideration to those of rank from that which was observed toward common people. It sometimes occurs that uncomfortable restraint may be best for the patient or that unpalatable draughts are indicated to bring about the desired effect, and the king was evidently apprehensive that the physician might, in the wish to save his royal master from the disagreeable measures, fail to secure efficiency in his treatment.

And while speaking of this there is perhaps no point upon which practitioners are more prone to err than by sacrificing energy of action for the agreeable taste of their remedies. By all means seek the most palatable of two medicines equally potent, but only in children should the acceptability to the taste influence a practitioner in cases of any gravity, and even with these force should be used when requisite.

Since the case referred to above, I have had occasion to render the same assistance to another lady in charge of one of the colleagues who had been in attendance there; and having observed by my deportment previously that there was no need of hurry or flurry to secure the desired end, I was permitted to take my own course, without interruption from him or from the family. He did not seem to consider that there was anything out of place in waiting to send to his office for a female catheter which was required to draw off the urine before any attempt was made to apply the forceps, and I was struck with the difference in the deportment of patient and all around, from having it impressed upon them, doubtless in advance, that it was not in keeping with my plan of doing things to have a whirl of excitement.

It may occur to some sober-sided colleague that these are very common-place points for the consideration of practitioners, yet the demeaning of oneself prudently under such circumstances has much to do with the exit of a case, and it should not be despised or neglected.

Having a due appreciation of the ordeal to be passed in assuming a place in the grand army that is marching on for the conquest of disease, it is still obligatory upon the recruits to be possessed of the requisite intellectual endowments and physical powers for the faithful and efficient performance of the duties which devolve upon those who enlist under the Hippocratic banner. It is a general belief that any blockhead may be a physician, and that any mechanic or even butcher may practice surgery successfully.

A father who had to determine upon the avocations suited to his five sons commenced by setting apart the one he thought most gifted to the study of law; the next in order of intelligence he made an engineer; the third in the scale he put to the trade of a blacksmith; the fourth-rate boy he concluded might preach the gospel; and the fifth, who was too stupid for anything else, was sent to a medical college to learn the art of curing the ills to which flesh is heir. Such is the ordinary view taken of the requisites for a doctor of medicine, and if we are to judge by the immense number of young men below the standard of mediocricy, who have entered the ranks in these latter years, we must infer likewise that the professors who decide upon the qualifications of the candidates think, with the father of the five sons, that any low order of mind with the most meagre attainments suffices to undertake the treatment of diseases; and it is, no doubt, a deliberate calculation that such may be allowed to kill or cure as a part of the grand programme of the survival of the fittest. If such outgrowths of a lax system of education and the pernicious competition among medical schools could be limited to the practice of homœopathy, and thus pursue an expectant course, letting the disease take its course, we might be reconciled to consider them as simple excrescences. But, unluckily, they have not discernment enough to know their own ignorance, and personify accurately the blind man with a club floundering about and giving blows indiscriminately which take effect, as it may be, in the destruction of the life or disease of the patient, with no sense of responsibility.

There was a period in the early history of the medical profession when scientific attainments availed little for their possessor in the competition for place in the confidence of the people, but with increased intelligence of the masses a distinction is now recognized between those who are well qualified and those not qualified for the respective departments of practice, and the individual who expects to succeed must fit himself properly for his work. That the medication of old women and quacks has a hold upon certain persons does not even create an exception to the general rule that thorough preparation is the best and surest recommendation of a medical man to the patronage of those around him. But it is thought by many that scholarship in other matters is not in any way required as a preliminary to entering upon the study of medicine, and if a young man has learned to read and write his own language moderately well, that he needs no other qualification for the comprehension of the teachings of the professors in the various branches of the medical schools. As it is recognized by all who understand the true aim of education that it is the proper training of the mind, it must be conceded that the intellect which has been cultivated confers great advantage over that which has been neglected, and, consequently, the student who has a fair preliminary education must attain to a better knowledge of a profession that requires an exercise of all the faculties, than one who is lacking such preparation. If any one, deluded with the idea at the outset that it is an easy matter to master anatomy, phyisiology, materia medica, chemistry, hygiene, etc., as preliminary to the comprehension of the more practical branches of his course, let him reflect how many men of prominence have devoted their best energies to the study and investigation of each of these, and yet have not reached the goal of their fond desire in the complete mastery of a single subject. It is very true that those who follow in the wake of the pioneer are free from much of the labor performed by the original investigation, and may, to a large extent, avail themselves of the results obtained. Yet the gatherer of the sheafs of the reaper has no small service to perform in tying them up and placing them in shocks, so as to be convenient for gathering in the harvest. Thus, the student who would turn to advantage the important work of those who have made valuable acquisitions in science must learn to arrange and systematize the data presented, so as to make them available for his future use. The human mind cannot serve the end of a mere sponge to soak up whatever it meets, but it must appropriate, as absorbents do in the body, the special qualities which are best adapted to nourish the different organs, and thus avail itself of the materials as its own, to be utilized in a fitting manner as occasion may require. To apply the intellectual energies to advantage, method is absolutely requisite, and this must be the effect of training, or, in other words, the individual who would study with benefit has to be taught how to study properly. To read and digest the substance of books, so as to receive nourishment for the mind, is the great end of study.

There are very few young men who have not had some mental training before entering a medical school that are in a condition to profit by the course of instruction given by the professors.

In the hushed silence of the amphitheatre in attendance upon the disquisitions of the teacher, if an attentive observer will glance around and note the physiognomy of the listeners, he may infer from the dull and blank stare of a portion of those occupying the seats that the words are as sounding brass and the tinkling cymbal to their ears, while others manifest an intelligent appreciation of all that is said. Thus it turns out, that those who commence without a beginning, never get a proper start in the cause, and never derive the profit which those do, who are better qualified by previous instruction to receive the instruction that is given. Such are like children born before their time, they are brought forth weak and puny, so that they are not capable of receiving proper nourishing from their mothers milk, and they continue feeble and rickety, reaching man's estate without the strength of muscle and firmness of the other organs of the body, which enables them to compete with their fellows in the struggle of life. The would-be-student of medicine who sets out without a proper foundation is an abortion and though he may be pronounced reliable by the professorial midwife, it would be better for society if he had not been kept alive by the artificial respiration and nursing which were extended by ill-judged charity.

The curriculum of most of the medical schools of good standing in the United States is so arranged as to impress general principles and if one comes for the study of special departments of practice, he will most assuredly be undeceived by his utter failure to comprehend what he realizes to be of prime necessity in a therapeutic and curative application of the means at his disposal. There is perhaps no branch of human knowledge in which a man requires more to learn first his ignorance than in the study of medicine; and woe be unto him who only comes to realize his deficiencics when he enters upon the practical duties of his profession. A young man without experience in the details of treating diseases, performing surgical operations, or managing difficult cases of labor, is very prone to consider that he has learned all that is requisite for practice in any of these departments; and it is only after encountering the obstacles which are daily presented in his adaptations of curative agencies to the varied causes which come under his observation, that he comes to understand how necessary it is to be diligent in analzying, comparing, and selecting the data which may have been collected in his course of study in the medical schools. There are few who have been activily engaged in practice for five years, that have not become thoroughly impressed with the conviction of the vast field to be explored to make them competent for all that devolves upon them, and at the end of ten years of zeal and application to service, the intelligent student of medicine has begun to reap real fruits from his unremitting attention to the teachings of others and his personal experience, so that now he learns more and faster than at any previous period of his professional pupilage. When he studies well the lessons before him until he has completed twenty years of active practice and close observation, he ought to be properly educated for the performance of his duties in the particular field which has been occupied. But should he have neglected the current literature of his profession, and contented himself to fall into the sluggish current, hemmed in by the dykes of prejudice and egotism, he is destined to run slowly down into the sea of oblivion, without obtaining the reward of diligence and of enterprise, which are to be reaped on the stream of progress. After thirty years of industrious investigation and searching after the prodromes and sequences of diseased action in the human system, the most learned of our profession have remained in uncertainty as to whence it cometh and whither it goeth, and have to content themselves with only plausible theories of the nature or essential constituents of specific disorders of the animal economy. The more ground that is explored, the greater is the zone of the unknown region beyond which comes into view, and research thus reveals to us what a vast field of investigation is opened up for the earnest study of the future explorers of the arena of medical science in all its phases.

With some sprightliness in boyhood, fostered by the faithful use of the birch to stimulate my faculties, I profited by a well-ordered academic course in youth, and received the advantages of the curriculum of a first class college previous to entering upon an office training with a preceptor. The daily recitation of lessons learned from the elementary text books were interspersed with a cautious drilling in the art of the manufacture of medicines, and witnessing occasionally a surgical operation. It strikes me that this good old regime has fallen somewhat into disuse in these latter days of hurrying up the early preliminaries of medical education, and the salutary office instruction is supplanted, to a large extent, by the cramming agency of a private lecturer who undertakes, for the gain of a few dollars from each of a club of students, to post them upon those points which are considered most essential for their comprehension of the regular lectures of the medical schools. The error of such a proceeding is palpable in crowding upon the student a multiplicity of subjects when his time and attention should be taken up with a few points well elaborated, so that they may be properly appropriated in going forward gradually from one step to another of his preparation. There are too many matters pressed upon the student's attention at one time by all the medical schools, and the effort to prolong the course so as to diminish the heterogeueous mass presented to his overburdened mind is certainly a move in the right direction. But the railroad speed of doing things now-a-days does not brook this delay in getting through the race for the title of M.D., and this helter skelter process of decking out a jackass with the skin of a lion can never succeed in hiding the ears that thrust themselves forth when the candidate is sent out with all the pomp and parade of graduation with a diploma.

The want of harmonious and general adoption of the extended course by all the schools in this country opens the way for a larger number of students to flock into those whose requirements are least, and as a consequence to abstract from the attendance upon the teaching of those where the standard is raised, so that, without any permanent endowment, the difficulty is almost insuperable for advancing the requirements of a third term of study. Were it practicable at an early day to enforce a law which should make it binding upon every medical college having a charter to exact attendance upon three graded courses of lectures, and not recognize the diploma of any school that departed from this programme, the competition would then become one of merit, and not that of pecuniary considerations as at present. The ordinary business rule that the demand regulates the supply has no place in the necessity for medical education among the fifty millions of people in this vast country, and, though the numbers that come forward to claim the title of M.D. have greatly increased during these latter years, this affords no sort of excuse for opening the door to the entrance of unworthy men into the medical profession.

Law is intended in every civilized government to protect the people against even their own vicious practices, and while State and county boards are enacting and executing measures to secure those sections against unqualified practitioners of medicine, the general government stands aloof. The preliminary requirements formerly insured a course of regular medical education. Thus, it was my good fortune, upon reaching manhood, to enter upon the practical duties of my profession with a fair average qualification for its requirements; yet now, in looking back through the subsequent pupilage of thirty-five years, it is a matter of profound astonishment with how little of real fitness for the responsible undertaking of a physician, that I then ventured upon the career of my profession. If another similar period could be allowed in advance of the present, with the moderate advancement I have been able to make by considerable practical observation with some reading and study upon passing events, I would hope to accomplish something towards the completion of my medical education by the use of more diligence and more intelligent adaptation of the limited resources with which I am now provided. Unfortunately, those who have been co-laborers during this prolific era are not permitted to secure the fruits of their past efforts any more than I am; and we must leave it to more vigorous successors to take up the echo of our voices and make it reverberate throughout all the dark valleys and over the craggy hills of scientific investigation, until the grand problems of prophylactic and sanitary regimen shall have been solved, and curative processes shall have been perfected.

Though many who have struggled against great difficulties during the last quarter of a century might reasonably calculate that the way has been paved for the march of progress in the next quarter, yet they can only put up land-marks to guide others on the way to distinction and honor.

If I were to risk a prediction as to the field in which the greatest triumphs may be obtained by future investigations, based upon what has already been done, I should expect the best fruits, from the more thorough comprehension of the nervous system, in a more judicious use of means for the correction of its disorders, and the general derangements of the functions dependent upon a deficient or vitiated nerve power to the different organs. It is true that some reforms have already been made, based upon the recognized importance of the role of the nervous system in various diseases not classified as nervous disorders; but if I am not entirely mistaken in the glimpses of light which have crept in from the rich physiological garner of facts respecting the intimate relations of the nerve fibrils and the capillaries, there is destined to be a grand revolution in nosology, pathology and therapeutics for those who enjoy the electric medical light which will usher in the twentieth century. That impressions made previously upon the nervous system have much to do in the development of disease is an established point, but the elucidation of the troubles which ensue from such impressions demands minute investigation, and the direction of measures for remedying these disorders is the grand desideratum of the present era. The whole armory of agencies calculated to modify the influence of the nerves on the physical organization requires to be overhauled and utilized in preventing and correcting injurious transmission through this channel, and electric phenomena as connected with atmospheric vicissitudes, which propagate hurtful impressions, should be studied carefully, as well as the application of this subtle fluid to the cure of diseases associated with the nervous system. How far this element of atmospheric electricity may influence hygiene appeals to the attentive consideration of men of science, and the common notion that a thunder storm operates favorably for the onset of epidemics may afford us a clue to an explanation of the general influence of electrical phenomena upon health.

The whole field of causative energies, that so insidiously bring trouble upon the physical organization, is involved in greater or less obscurity, and presents its sullen exaction for elucidation. Though the given theory of the origin of many diseases has been entertained by a large part of the medical profession, there is such skepticism manifested by some competent observers as to shake the faith of many in this hypothesis and lead to its abandonment.

In the surgical branch, much has been gained, but there remains a grand field for exploration ; and the complications of shock and septicæmia call for patient investigation by pathologists.

Important as have been the advances in all departments of practice, they have their chief significance in furnishing the ground-work for other researches that are decidedly indicated for placing the medical profession on the true basis of well-defined principles and of ascertained results as a science.

That each investigator shall avail bimself of the data accumulated for exploring the yet hidden mysteries of nature and disease, so as to reach a satisfactory solution of the great curative problem, is the consummation devoutly to be wished by the profession.

The speculative theories of the novelist in like manner as the effete applications of the antiquarian conservatist, must give way to the searching scrutiny of practical investigation, and the tendency which has been manifested on the part of some to see nothing worthy of consideration which has not the sanction of great names, is destined to be corrected by the dogged persistence of faithful laborers in all the departments of clinical observation. The recognition of the improvements in gynæcology must serve as a stimulus to those engaged in other branches of practice, and though experience has already proved that rarities are not to be adopted too hastily, the initiative spirit of the age has conferred many benefits upon the grand total of therapeutics. There is no delusion, however, so well calculated to undermine genuine progress in science, as the grasping after those modes of accomplishing results which are different from the ordinary well-tried plans of treatment, whether in the practice of medicine or surgery; and it strikes me most forcibly that our young men especially, ought to have it impressed upon them, to hold fast to that which is good and trustworthy in the experience of those who have gone before them. A safe and efficient application of means for the relief of any disorder should never be discarded for that which come with a flourish of trumpets because it is new, and it behooves every practitioner to be well posted in all that is established upon a solid foundation, so that he shall be able at all times to give answer for not being led over bogs and moors in pursuit of some will-o'-the-wisp that flitters up before him.

I would not be understood as objecting to anything because it is new or rare, and whether the recent developments respecting the microbia and crypta syphitica are entitled to all the confidence which their discovers claim for them or not, we should not reject them because of their comparative novelty; and yet we should not be led into the error of accepting a thing because it is new, without full and satisfactory demonstration of its utility. This is the touchstone to be applied to all candidates for our favor, and unless a reason presents self-evident proofs of adaptability to the purpose for which it is recommended, it should be finally tested before adopting it in practice. There is getting to be quite a taste for curiosities in medical literature, and if a Disraeli were to cull from the current items in reports of rare cases, unique specimens, etc., which figure in our medical journals now-a-days, it might seem as a kaleidoscope to amuse amateurs, yet it would be of no more practical utility than that unstable variety of colors. As monstrosities prove nothing as to the regular organization, so then striking peculiarities of degeneration gives us no instruction in the ordinary development of diseased action, and it is no more warrantable in a faithful picture of the progress of medical science to introduce those anomalies, than it is becoming in the work of fiction to permit scenes which have no counterpart in nature. That which is exceptional cannot be made to prove the rule in physiology or pathology, and it is to be desired that this penhant for the *outre* in medical literature shall be displaced by a correct appreciation of the useful and practical results of observation.

