# Errata

**Published:** 1883-07

**Authors:** 


### ERRATA.

Our readers will please note the following corrections in the article of Dr. Gaston, which appeared in the February number of the REGISTER:

On page 263, 25th line from the top, for "gain" read "give." On same page, 27th line from top, for "depressed" read "depraved."

On page 272, 8th line from bottom, for "reliable" read "viable."

On page 273, 14th line from top, for "causes" read "cases."

On page 277, 19th line from top, for "onset" read "arrest." On same page, 15th line from bottom, for "given" read "germ."

On page 279, 4th line from top, for "reason" read "measure."

